# Magnetic Field Sensing Based on Magnetic-Fluid-Clad Multimode-Singlemode-Multimode Fiber Structures

**DOI:** 10.3390/s141019086

**Published:** 2014-10-14

**Authors:** Jiali Tang, Shengli Pu, Shaohua Dong, Longfeng Luo

**Affiliations:** College of Science, University of Shanghai for Science and Technology, Shanghai 200093, China; E-Mails: tangjiali1230@gmail.com (J.T.); zhuiguang0903@163.com (S.D.); longfengllf@163.com (L.L.)

**Keywords:** magnetic fluid, multimode-singlemode-multimode fiber structure, mode interference

## Abstract

Magnetic field sensing based on magnetic-fluid-clad multimode-singlemode-multimode fiber structures is proposed and experimentalized. The structures are fabricated out using fiber fusion splicing techniques. The sensing principle is based on the interference between the core mode and cladding modes. Two interference dips are observed in our spectral range. Experimental results indicate that the magnetic field sensing sensitivities of 215 pm/mT and 0.5742 dB/mT are obtained for interference dip around 1595 nm. For interference dip around 1565 nm, the sensitivities are 60.5 pm/mT and 0.4821 dB/mT. The response of temperature is also investigated. The temperature sensitivity for the dip around 1595 nm is obtained to be 9.93 pm/°C.

## 1. Introduction

Magnetic fluid (MF) is a kind of stable colloidal suspension system consisting of surfactant-coated magnetic nanoparticles with typical sizes of 10 nm dispersed in a suitable liquid carrier. It possesses both the features of magnetic property of solid magnetic materials and fluidity of liquids, which makes it an attractive material. MF has many unique magneto-optical effects such as linear dichroism, linear birefringence, Faraday rotation and Faraday ellipticity. The magneto-optical properties of MFs imply their various applications in optical and sensing fields, such as magnetic field sensors [1–9], slowing light [10], optical switches [11] and modulators [12]. One of the major applications is magnetic field sensing with optical techniques. On the other hand, due to the advantages of compact structure, high precision and resolution, fiber-optic structures have been widely used in many applications. Therefore, the fiber-based magnetic field sensing using MF as the sensitive material is getting continuing interest recently [13–15].

Multimode-singlemode-multimode (MSM) fiber structures have been successfully utilized to sense temperature [16] and bend [17]. In this work, the MSM structure combining with MF is employed for magnetic field sensing, which has the advantages of low cost and ease of fabrication. In other reported works, considerably complicated techniques are usually involved, such as tapering [3,6], corroding [5], core-offset fusion splicing [6] and microfabrication [13]. In our work, the simple fusion splicing technique is used. The high-order modes are excited in the singlemode fiber (SMF) cladding and then interfere with the core mode. The effective refractive indices of the involved cladding modes can be influenced by the external environment. Then, the sensing purpose is achieved.

## 2. Experiments and Principles

**Figure 1.**
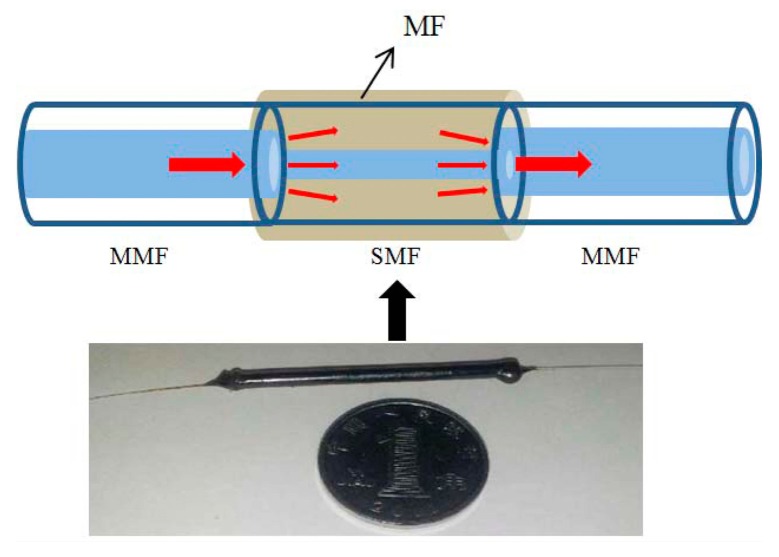
Schematic of the multimode-singlemode-multimode (MSM) structure. The low panel shows the experimental structure.

The proposed MSM structure consisting of a segment of SMF sandwiched between two sections of multimode fibers (MMFs), which is illustrated in Figure 1. The MMFs act as the lead-in and lead-out fibers, respectively. The core and cladding diameters of the MMF are 105 and 125 μm, respectively. The length of SMF is 30 mm. The as-fabricated MSM structure is put into the capillary filled with MF. The inner diameter of the capillary is about 2 mm. Both ends of the capillary are sealed to avoid MFs leaking or evaporating. In our experiments, the water-based Fe_3_O_4_ MFs with saturation magnetization of ∼20 mT and density of 1.18 × 10^3^ kg/m^3^ are provided by Beijing Sunrise Ferrofluid Technological Co., Ltd. The diameter of the magnetic nanoparticles within the MFs is around 10 nm.

**Figure 2.**
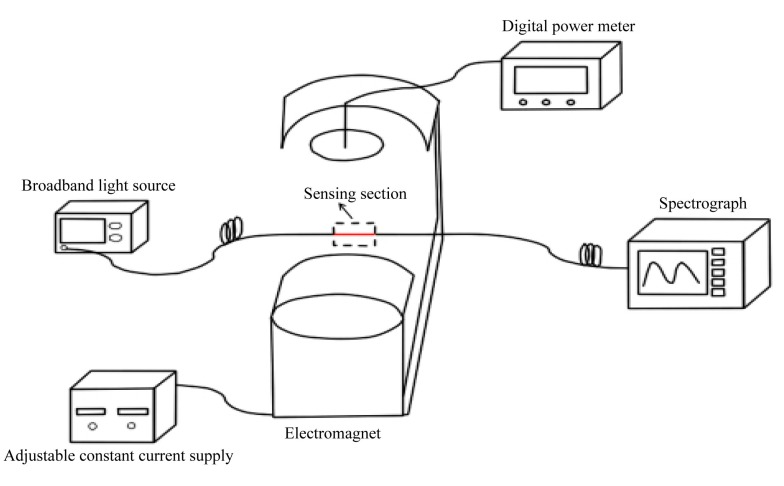
Experimental setup for investigating the magnetic field sensing properties of the MSM structure.

The experimental setup for investigating the magnetic field sensing properties of the MSM structure is schematically shown in Figure 2. The strength of the magnetic field can be tuned by adjusting the supply current. The light from the broadband light source (BBS) is coupled into the lead-in MMF and then approaches the SMF. Due to the large mode field mismatch, the lead-in MMF plays the role of mode coupler to couple the fundamental core mode of the lead-in MMF into the core and cladding modes of the sensing SMF. The interference between the core and cladding modes occurs in the lead-out MMF and is transmitted into the optical spectrometer analyzer (OSA). The transmission spectrum can be analyzed by using the two-mode interference model for simplification [18,19]

(1) $${\text{I}}_{\text{out}}(\lambda )={\text{I}}_{\text{core}}(\lambda )+{\text{I}}_{\text{clad}}(\lambda )+2\cos(2\pi \Delta \text{nL}/\lambda )$$

where I_core_ and I_clad_ are the intensities of the fundamental mode and high-order cladding modes, respectively. Δn is the effective refractive index difference between the core mode and cladding mode. L is the effective length of the sensing section, which equals the length of SMF sandwiched between the MMFs in our experiments. According to Equation (1), the interference valley wavelength λ_m_ can be described as

(2) $$\lambda_{\text{m}}=2\Delta \text{nL}/(2\text{m}+1)$$

where m is the interference order.

The effective refractive index of the core mode is unaffected but the cladding modes will be influenced by the environmental refractive index change. So the effective refractive index difference between the core and cladding modes will vary with the environmental refractive index, which will result in the shift of transmission spectrum according to Equation (1). Besides, the cladding mode energy leakage will vary with the mode effective refractive index difference. Therefore, the transmission loss of the sensing structure will change with the environmental refractive index as well. It is well known that MFs have unique properties of magnetic-field-dependent refractive index [20–22]. When MFs are used as the cladding of the sensing section, the interference valley wavelength and transmission loss may change with the magnetic field, which can be employed for magnetic field sensing.

## 3. Results and Discussion

The principle of the magnetic field sensing of the proposed structure is based on the refractive index sensing in nature. Hence, the sensing properties of the MSM structure in a wide range of refractive index variation will be characterized first. The magnetic field sensing properties are then investigated using a specific MF as the cladding of the MSM structure. To obtain various liquids with different refractive indices, the glycerinum liquids with different concentrations are prepared. The refractive indices of the as-prepared glycerinum liquids are listed in Table 1.

**Figure 3.**
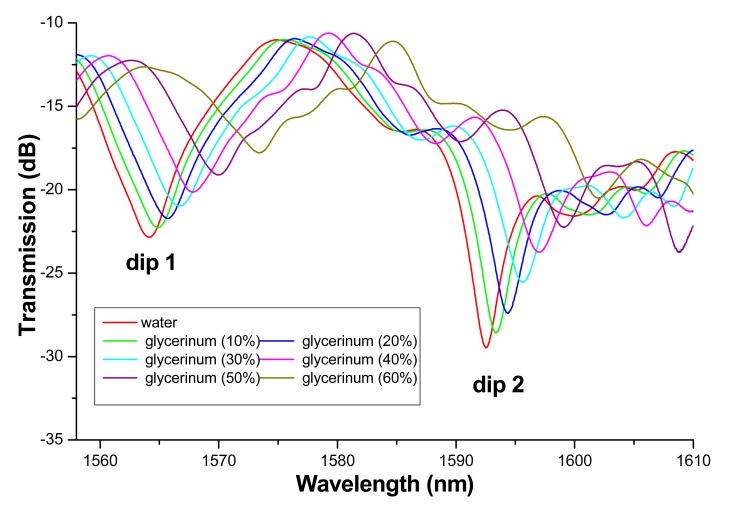
Transmission spectra of the proposed sensing structure surrounded with glycerinum liquids of different concentrations.

The typical transmission spectra of the sensing structure surrounded with glycerinum liquids of different concentrations are shown in Figure 3. Two distinct interference valleys are observed in the wavelength range of 1560 to 1610 nm. The interference valley around 1565 nm is referred as dip 1 and that around 1595 nm is referred as dip 2. Figure 3 indicates that both of the interference valleys shift to long wavelength side with the refractive index increase of the surrounded liquids. In our experiment, the refractive index of SMF is constant and larger than those of the liquids surrounding the SMF. So, most of the mode energy is confined inside the SMF and then the effective refractive indices of the cladding modes are mainly related with the variation of mode energy inside the SMF. With the refractive index increase of surrounding liquid, the cladding mode energy inside the fiber will decrease, which will result in the decrease of effective refractive index of the cladding mode. Therefore, Δn will increase with the refractive index increase of surrounding liquid. This will lead to the red-shift of the wavelength valley according to Equation (2). In addition, the depth of the interference valley decreases with the refractive index increase. It may be assigned to the relatively large field outside the fiber for the higher refractive index case and then the loss due to the external environment increases and the interference effect is weakened.

Figure 3 indicates that the wavelength and intensity of the interference dip are sensitive to the refractive index of the environmental liquids. It is well known that the refractive index of MF can be influenced by a magnetic field. Hence, magnetic field sensing may be realized when using MF as the cladding of the MSM structure.

**Figure 4.**
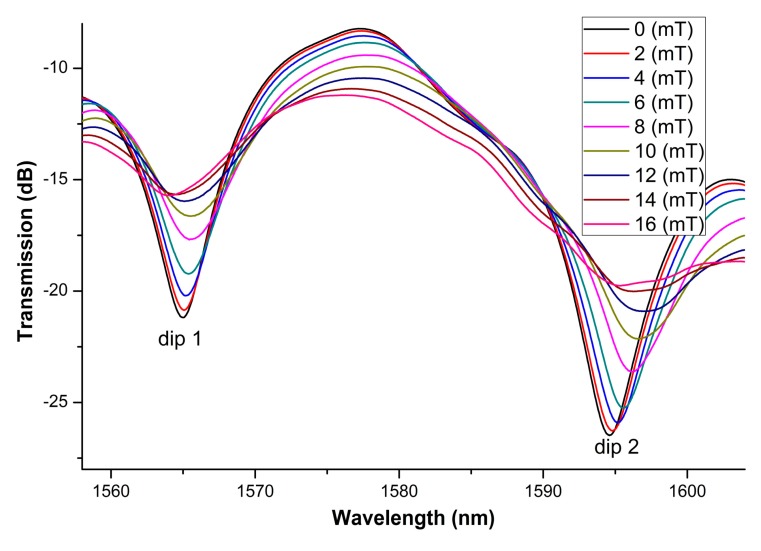
Transmission spectra of the proposed sensing structure at magnetic field strength ranging from 0 to 16 mT.

**Figure 5.**
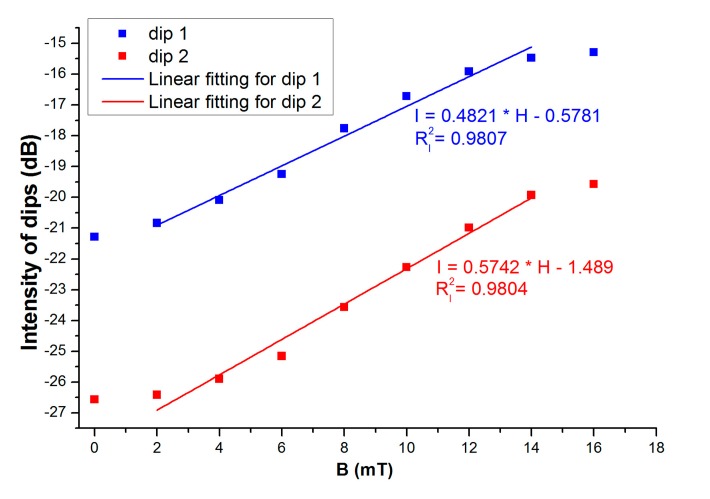
Intensities of dip 1 and dip 2 as functions of magnetic field.

The transmission spectra of the sensing structure at magnetic field strength ranging from 0 to 16 mT are described in Figure 4. The corresponding intensities of the interference valleys as functions of magnetic field are plotted in Figure 5. From Figures 4 and 5, we can find that the depths of the interference valleys decrease with the magnetic field monotonously. With the magnetic field increases, the interference valleys become shallower and shallower. Then, the tendency of saturation is achieved. These are assigned to the unique properties of magnetic-field-dependent refractive index and loss of MF. The maximum magnetic field sensing sensitivities of 0.4821 dB/mT and 0.5742 dB/mT can be obtained for dips 1 and 2, respectively. The sensitivities for dips 1 and 2 are different. The possible reason is that the interference between different modes results in dips 1 and 2, respectively. Different modes have different sensitivities to the external surrounding [23]. The proposed sensing structure is immersed in MF. The refractive index of MF would be changed with the change of external magnetic field. For the wavelength interrogation, the sensitivity of the proposed structure is higher (215 pm/mT) than that of [5] (−168.6 pm/mT). For the intensity interrogation, the sensitivity of the proposed structure is higher (0.5742 dB/mT) than the highest sensitivity of [6] (0.3407 dB/mT). Compared to [8], the proposed structure is simple and cost-effective. Zheng *et al.* [8] used a tilted-fiber Bragg grating cascaded with a chirped-fiber Bragg grating.

**Figure 6.**
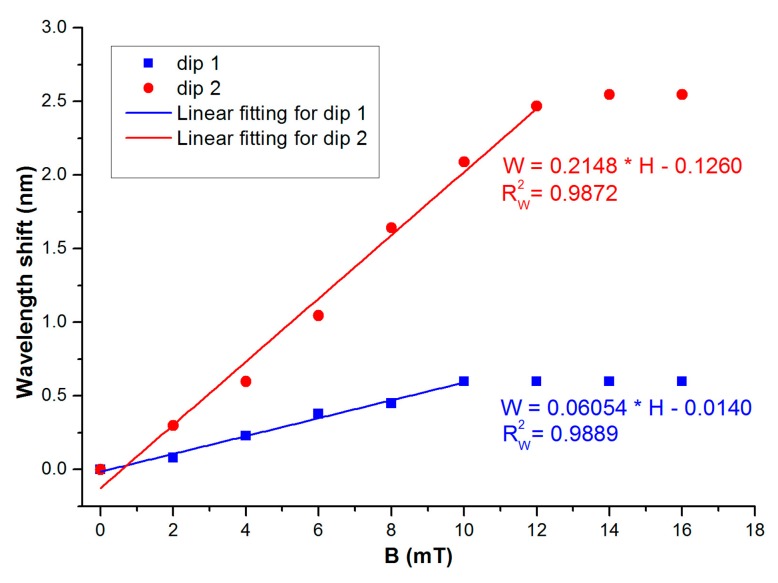
Wavelengths of dips 1 and 2 change with magnetic field.

The dip wavelength as a function of external magnetic field is shown in Figure 6, which displays a linear dependence at low field regime. Figures 5 and 6 indicate that a good linear relationship is found at moderate magnetic field strength. These are due to the unique magneto-optical properties of MFs. Therefore, the linear fitting is only made at a moderate magnetic field region. Besides, linear relationship is favorable for practical sensing application from the point of interrogation. The linear fitting method has also been employed by other authors (for example, see [5,6]). The sensitivities are obtained to be 60.5 pm/mT and 215 pm/mT for dips 1 and 2, respectively.

**Figure 7.**
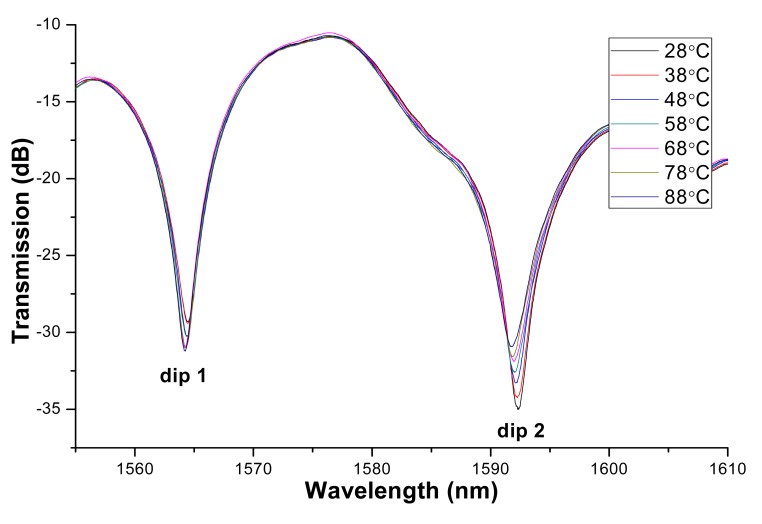
Transmission spectra of the proposed sensing structure at different temperatures.

**Figure 8.**
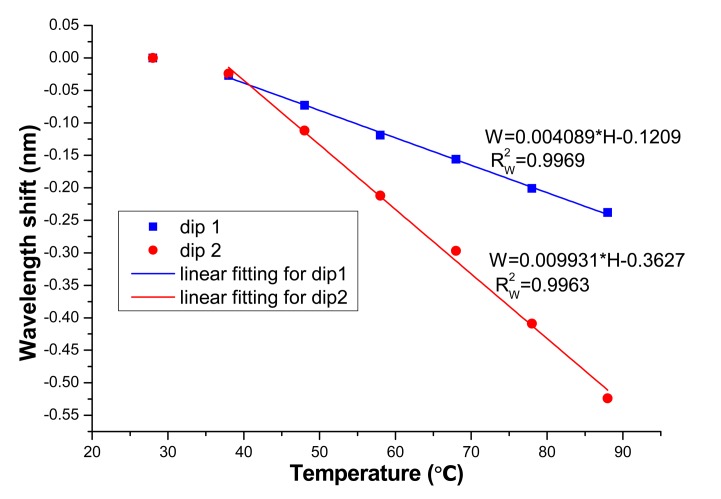
Wavelength of dips 1 and 2 change with temperature.

To study the effect of temperature on the sensing performance of the proposed MSM structure, the experiments are conducted at various temperatures. The corresponding results are described in Figures 7 and 8. Figures 7 and 8 show that the dip wavelength blue-shifts with temperature. The refractive index of MF decreases with temperature. Consequently, Δn will decrease with temperature, which will result in the blue-shift of the wavelength valley. From Figure 8, the maximum sensitivity of temperature effect is obtained to be 9.93 pm/°C (for dip 2). So for some applications (e.g., temperature variation is remarkable or high precision magnetic field measurement), temperature calibration is needed.

## 4. Conclusions

In summary, a kind of magnetic field sensor based on MF-clad SMF sandwiched between two pieces of MMFs is proposed. It was found that the depth of the interference valley varies with the magnetic field strength. Meanwhile, the interference valley wavelengths change with the external magnetic field monotonously. The corresponding sensitivities are obtained to be 0.4821 dB/mT and 60.5 pm/mT for the wavelength dip around 1565 nm. For the wavelength dip around 1595 nm, the sensitivities are 0.5742 dB/mT and215 pm/mT. The temperature effect of the sensing structure is also investigated. The temperature sensitivity can reach 9.93 pm/°C. The proposed sensing structure has the advantages of low-cost and compactness.

## Figures and Tables

**Table 1.** Refractive index of glycerinum liquids with different concentrations.

**Liquids**	**Water**	**Glycerinum Liquids with Different Concentrations**					

		**10%**	**20%**	**30%**	**40%**	**50%**	**60%**
RI	1.3300	1.3448	1.3575	1.3707	1.3841	1.3909	1.4123
